# Temporal Trends in Parkinson’s Disease Related Mortality from 1999-2020: A National Analysis

**DOI:** 10.3310/nihropenres.13623.1

**Published:** 2024-09-13

**Authors:** Balamrit Singh Sokhal, Sowmya Prasanna Kumar Menon, Thomas Shepherd, Sara Muller, Amit Arora, Christian Mallen

**Affiliations:** 1 School of Medicine, Keele University, Keele, England, UK; 2 Department of Geriatrics, University Hospital North Midlands, Stoke-On-Trent, UK

**Keywords:** Parkinson’s Disease; Mortality; Outcomes; Epidemiology.

## Abstract

**Introduction:**

Parkinson’s disease (PD) is the most common neurodegenerative movement disorder and is associated with significant disability. The prevalence is rising, and studies have reported potential sex and race disparities in patient outcomes. Data about the demographic trends in PD-related mortality in the United States (US) is limited. This descriptive study aimed to report the national demographic trends in PD-related mortality over a 20-year period.

**Methods:**

The US Centers for Disease Control and Prevention Wide-Ranging Online Data for Epidemiological Research (CDC-WONDER) Underlying Cause of Death database from January 1999 to December 2020 was used to determine the PD-related age adjusted mortality rate (AAMR) stratified by age, sex, ethnicity and geographic area, with the 1999 deaths as the reference group. Annual percentage change (APC) for AAMR was then calculated using Joinpoint regression.

**Results:**

There were 515,884 PD-related deaths in the study period. The AAMR increased from 5.3 per 100,000 population in 1999 to 9.8 per 100,000 in 2020. Males had consistently higher AAMR than females and white race had consistently higher overall AAMR (7.6 per 100,000), followed by American Indians/Alaska Natives (4.4 per 100,000), Asians/Pacific Islanders (4.1 per 100,000) and Black/African Americans (3.4 per 100,000). The Midwest had the highest AAMR followed by West, South and Northeast. Utah, Idaho and Minnesota had the highest state-level AAMR.

**Conclusions:**

This study identified significant age, sex, race and geographic disparities in PD-related mortality in the US. Older age, male sex, white race and Midwest locality were associated with the highest AAMR.

## Plain Language Summary

Parkinson's disease (PD) is a common disease that impacts the brain and as a result, movement. One in 37 people will be diagnosed with PD in their lifetime, and the risk of developing PD increases with age. PD can lead to variety of complications such as falls, depression, and death. This research aimed to summarise the demographic trends in PD-related death in the United States from 1999–2020 using death certificate data. There were 515,884 PD-related deaths in the study period. Death rates increased from 5.3 per 100,000 population in 1999 to 9.8 per 100,000 in 2020. In terms of demographics, firstly, male sex had a higher rate than female sex. Secondly, white race had death rate than American Indians/Alaska Natives, Asians/Pacific Islanders and Black/African Americans, Finally, the Midwest had a higher death rate than the West, South and Northeast. These results could be because PD is more common in these groups. Future research could investigate underlying reasons behind disparities in mortality. In summary, PD is a significant cause of death in the US and there are differences in demographic trends in PD-related deaths. The number of deaths from PD per population count is higher with older ages, in males, people of white race and in the Midwest. Knowledge of these trends is important to build healthcare services around this growing population.

## Introduction

Parkinson’s Disease (PD) is the most common neurodegenerative disorder and is characterised by resting tremor, rigidity and bradykinesia. It is a leading cause of disability worldwide ^1– 3^. The prevalence of PD is rising with the increasing average age of the population, with a prevalence of 1903 per 100,000 in over 80 year olds compared to 41 per 100,000 in 40 to 49 year olds ^4^. Studies suggest geographic variation, North America having the highest non-age adjusted prevalence (1,601 per 100,000) and Asia having the lowest prevalence (646 per 100,000), possibly due to genetic susceptibility ^4, 5^, as well as the differing age profiles of the populations. The prevalence of PD is almost double in males compared to females, possibly due to a protective effect of female gonadal factors and the lack of a protective effect of male gonadal factors ^6, 7^. Despite this, existing studies report increased mortality in females ^6, 7^. Worldwide non-age adjusted mortality rate from PD has increased between 1994 and 2019 (1.76 per 100,000 in 1994 and 5.67 per 100,000 in 2019), which could be due to a variety of factors such as an ageing population and improved reporting ^3^. In addition to PD being associated with a significant individual burden, PD is also associated with large economic burden ^3^. In the United States (US) in 2017, there were 1 million people living with PD, incurring a total economic cost of $51.9 billion of direct and indirect healthcare costs, which is projected to increase substantially ^8^.

There is a paucity of published data about the annual demographic trends in PD-related mortality in the US ^3^. Therefore, the aim of this study was to examine the demographic trends in PD-related mortality using national data. Knowledge of these trends is important to monitor the growing population living with PD and improve end of life care services.

## Methods

### Patient and Public Involvement

There was no public or patient involvement in this study.

The Centers for Disease Control and Prevention Wide-Ranging OnLine Data for Epidemiologic Research (CDC-WONDER) underlying cause of death database was utilised from 1999 to 2020. CDC-WONDER is a dataset containing details on the cause of death from the 50 US states and the District of Columbia, as obtained from death certificates ^9^. More than 99% of US deaths are recorded on CDC WONDER ^9^. Several studies have previously used this dataset to determine the trends in mortality for chronic conditions as well as the incidence and mortality of different cancers ^10– 13^. Using the International Classification of Diseases- Tenth Revision (ICD-10) code for PD (G20), data were collected on records with PD stated as the underlying cause of mortality ^14^.

Data for year, population size, demographics (age at death, sex and race), location of death (outpatient, emergency room, inpatient, death on arrival or unknown), region and state were extracted from CDC-WONDER using death certificate information.

Annual PD-related crude mortality rates (CMR) and age-adjusted mortality rates (AAMR) per 100,000 persons were determined. To calculate crude mortality rate, the number of PD-related deaths was divided by the population of the given year and presented with 95% confidence intervals (CI). AAMR was calculated by standardizing the PD-related deaths to 1999 US population and presented with 95% CI ^15^. Joinpont regression was used to quantify annual national trends in PD-related mortality by calculating the annual percentage change (APC) using the Joinpoint software ^16^. Joinpoint regression identifies significant differences in AAMR over time using log-linear regression models for temporal variations. Microsoft Excel was used to visually present data ^17^.

This study did not require ethical approval from an institutional review board. CDC-WONDER is an publicly available, anonymised dataset ^9^. Therefore, this study was performed in accordance with the ethical standards laid down in an appropriate version of the 1965 Declaration of Helsinki. This study was reported in accordance with the STrengthening the Reporting of Observational studies in Epidemiology (STROBE) guidelines ^18^.

## Results

### Annual Trends for PD-Related Mortality

**Figure 1.**
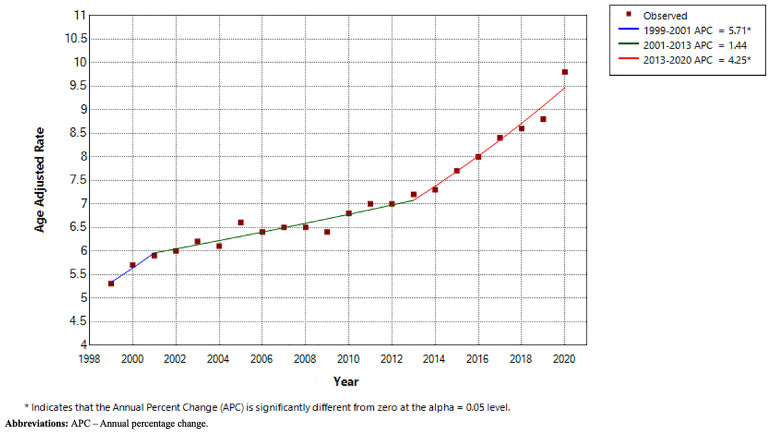
Joinpoint regression analysis by year.

Between 1999 to 2020, there were a total 515,884 PD-related deaths. Overall CMR was 7.6 per 100,000 persons (95% CI 7.6-7.7) and AAMR was 7.1 per 100,000 persons (95% CI 7.1-7.2). CMR was 5.2 per 100,000 in 1999 (95% CI 5.1-5.3) and 12.1 per 100,000 in 2020 (95% CI 12.0-12.3). AAMR was 5.3 per 100,000 in 1999 (95% CI 5.2-5.4) and 9.8 per 100,000 in 2020 (95% CI 9.7-9.9). Using Joinpoint regression analysis, the APC in AAMR was 5.71 (95% CI 2.00-8.67) from 1999–2001, 1.44 (95% CI -0.07-1.85) from 2001–2013 and 4.25 (95% CI 3.34-5.62) from 2013–2020 (shown in Table 1 and Figure 1).

**Table 1.** Demographic trends in mortality by year.

	Variables	Year																						
		**1999**	**2000**	**2001**	**2002**	**2003**	**2004**	**2005**	**2006**	**2007**	**2008**	**2009**	**2010**	**2011**	**2012**	**2013**	**2014**	**2015**	**2016**	**2017**	**2018**	**2019**	**2020**	**1999–** **2020**
**Overall data**	**Number of deaths**	14511	15591	16466	16857	17890	17898	19427	19431	19913	20320	20391	21835	22978	23664	25016	25972	27793	29494	31754	33596	35073	40014	515884
	**Population (million)**	2.8	2.8	2.9	2.9	2.9	2.9	3.0	3.0	3.0	3.1	3.1	3.1	3.1	3.1	3.2	3.2	3.2	3.2	3.3	3.3	3.3	3.3	6,746.4
	**CMR ([95%CI]**	5.2 [5.1- 5.3]	5.5 [5.5- 5.6]	5.8 [5.7- 5.9]	5.9 [5.8- 5.9]	6.2 [6.1- 6.3]	6.1 [6.0- 6.2]	6.6 [6.5- 6.7]	6.5 [6.4- 6.6]	6.6 [6.5- 6.7]	6.7 [6.6- 6.8]	6.6 [6.6- 6.7]	7.1 [7.0- 7.2]	7.4 [7.3- 7.5]	7.5 [7.4- 7.6]	7.9 [7.8- 8.0]	8.1 [8.0- 8.2]	8.6 [8.5- 8.7]	9.1 [9.0- 9.2]	9.7 [9.6- 9.9]	10.3 [10.2- 10.4]	10.7 [10.6- 10.8]	12.1 [12.0- 12.3]	7.6 [7.6- 7.7]
	**AAMR [95%CI}**	5.3 [5.2- 5.4]	5.7 [5.6- 5.7]	5.9 [5.8- 6.0]	6.0 [5.9- 6.0]	6.2 [6.1- 6.3]	6.1 [6.1- 6.2]	6.6 [6.5- 6.6]	6.4 [6.4- 6.5]	6.5 [6.4- 6.6]	6.5 [6.4- 6.6]	6.4 [6.3- 6.5]	6.8 [6.7- 6.9]	7.0 [6.9- 7.0]	7.0 [6.9- 7.1]	7.2 [7.1- 7.3]	7.3 [7.3- 7.4]	7.7 [7.6- 7.8]	8.0 [7.9- 8.1]	8.4 [8.3- 8.5]	8.6 [8.5- 8.7]	8.8 [8.7- 8.9]	9.8 [9.7- 9.9]	7.1 [7.1- 7.2]
**Age**	**45-54 CMR**	0.1	0.1	0.1	0.1	0.1	0.1	0.1	0.1	0.1	0.1	0.1	0.1	0.1	0.1	0.1	0.2	0.1	0.2	0.1	0.2	0.2	0.2	-
	**55-64 CMR**	1.0	1.1	1.2	1.2	1.2	1.2	1.4	1.2	1.1	1.1	1.3	1.3	1.3	1.4	1.4	1.4	1.5	1.6	1.7	1.7	1.8	2.0	-
	**65-74 CMR**	10.9	11.4	11.6	12.1	12.5	11.8	12.7	12	11.6	12.1	11.1	11.7	12.6	12.2	12.7	12.9	13.7	14.1	15.6	16	16.6	18.8	-
	**75-84 CMR**	57.9	61.6	64.3	63.4	67.3	67.1	70.7	69.2	71.1	70.7	70.3	74.3	75.7	75.8	78	78.8	81.9	85	89.1	90.5	92.8	104.7	-
	**Over 85 CMR**	123.7	131.2	136.4	141.4	144.9	144.3	155.4	156.9	156.2	156.6	156.2	164.7	167.6	171.4	177.2	181.1	189.7	197.7	205.1	213.5	214.5	236.2	-
**Sex**	**Number of deaths**	8,221	8,751	9,361	9,540	10,120	10,311	11,172	11,214	11,472	11,864	11,986	12,754	13,459	14,033	14,978	15,571	16,761	17,741	19,252	20,371	21,443	24,315	-
	**Male AAMR**	8.4	8.8	9.2	9.2	9.5	9.5	10.1	9.9	9.9	10	9.9	10.3	10.5	10.6	11	11.1	11.6	11.9	12.5	12.8	13.0	14.4	-
	**Number of deaths**	6,290	6,840	7,105	7,317	7,770	7,587	8,255	8,217	8,441	8,456	8,405	9,081	9,519	9,631	10,038	10,401	11,032	11,753	12,502	13,225	13,630	15,699	-
	**Female AAMR**	3.6	3.9	4.0	4.1	4.3	4.2	4.5	4.4	4.4	4.4	4.3	4.6	4.7	4.6	4.8	4.8	5.0	5.3	5.5	5.7	5.8	6.5	-
**Race**	**Number of deaths**	13,810	14,853	15,686	16,036	16,944	16,959	18,387	18,395	18,731	19,184	19,253	20,607	21,562	22,213	23,392	24,132	25,761	27,292	29,230	30,845	32,205	36,631	-
	**White AAMR**	5.6	6.0	6.2	6.3	6.6	6.5	7.0	6.9	6.9	6.9	6.9	7.3	7.4	7.5	7.7	7.8	8.2	8.6	9.0	9.2	9.4	10.5	-
	**Number of deaths**	502	532	546	555	624	622	705	673	791	731	717	795	881	918	1,031	1,149	1,183	1,367	1,527	1,688	1,667	2,075	-
	**Black or African** **American AAMR**	2.4	2.5	2.5	2.5	2.8	2.8	3.0	2.8	3.3	2.9	2.8	3.0	3.2	3.3	3.5	3.8	3.8	4.2	4.5	4.8	4.6	5.5	-
	**Number of deaths**	173	161	198	213	263	273	270	307	336	337	365	371	458	470	524	607	751	749	875	938	1,074	1,173	-
	**Asian or Pacific** **Islander AAMR**	3.4	2.9	3.2	3.3	3.8	3.7	3.4	3.7	3.8	3.5	3.7	3.5	4.0	3.9	4.0	4.3	4.9	4.7	5.0	5.1	5.6	5.8	-
	**Number of deaths**	26	45	36	53	59	44	65	56	55	68	56	62	77	63	69	84	98	86	122	125	127	135	-
	**American Indian or** **Alaska Natives** **AAMR**	2.8	4.1	3.0	4.4	4.9	3.6	4.8	4.2	3.9	4.7	3.3	3.8	4.2	3.4	3.4	4.0	4.3	3.4	4.9	4.7	4.5	4.3	-

**Abbreviations:** AAMR – Age adjusted mortality rate; CI – Confidence interval; CMR – Crude mortality rate; PD – Parkinson’s Disease; SE – Standard error.

### Annual trends for PD-related mortality stratified by age

**Figure 2.**
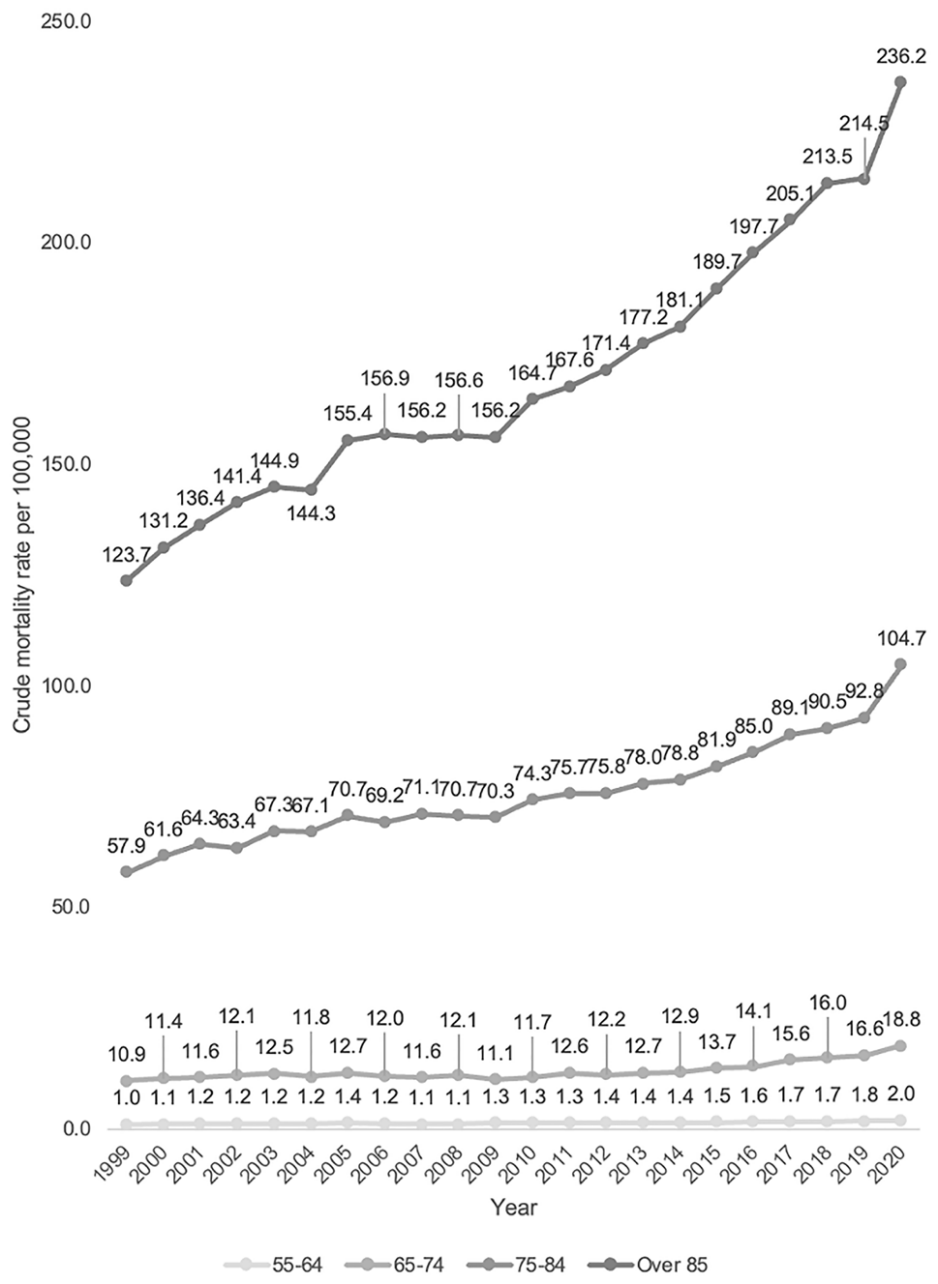
Crude mortality rate from 1999–2020 stratified by age.

**Figure 3.**
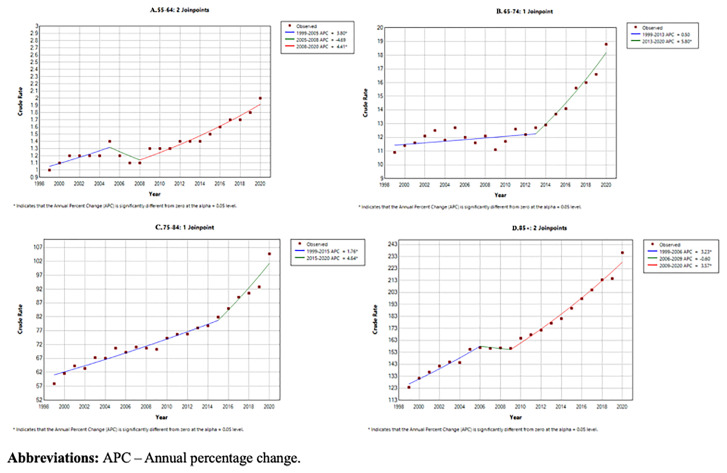
Joinpoint regression analysis by year stratified by 10 year age categories: **A**. 55–64, **B**. 65–74, **C**. 75–84, **D**. Over 85.

Older age categories had a consistently higher CMR throughout the 20 year period ( Table 2 and Figure 2). CMR for patients aged over 85 was 7.6 (95% CI 172.8-174.3), compared to a CMR of 1.4 (95% CI 1.4-1.4) for patients aged 55–64 (shown in Figure 3).

**Table 2.** Mortality rate stratified by Census region, Census division and HHS region.

	Variables	AAMR [95%CI]
Census region	Census Region 1: Northeast	6.2 [6.1-6.2]
	Census Region 2: Midwest	7.7 [7.6-7.7]
	Census Region 3: South	7.2 [7.1-7.2]
	Census Region 4: West	7.4 [7.4-7.5]
Census division	Division 1: New England	6.8 [6.7-6.9]
	Division 2: Middle Atlantic	6.0 [5.9-6.0]
	Division 3: East North Central	7.5 [7.5-7.6]
	Division 4: West North Central	8.0 [7.9-8.1]
	Division 5: South Atlantic	7.0 [6.9-7.0]
	Division 6: East South Central	7.2 [7.1-7.3]
	Division 7: West South Central	7.5 [7.5-7.6]
	Division 8: Mountain	8.1 [8.0-8.2]
	Division 9: Pacific	7.1 [7.1-7.2]
HHS region	HHS Region #1	6.8 [6.7-6.9]
	HHS Region #2	5.3 [5.2-5.3]
	HHS Region #3	7.2 [7.1-7.2]
	HHS Region #4	7.0 [6.9-7.0]
	HHS Region #5	7.7 [7.6-7.7]
	HHS Region #6	7.6 [7.5-7.7]
	HHS Region #7	7.8 [7.7-7.9]
	HHS Region #8	8.1 [8.0-8.2]
	HHS Region #9	6.9 [6.9-7.0]
	HHS Region #10	8.4 [8.3-8.5]

Abbreviations: AAMR – Age adjusted mortality rate; CI – Confidence interval; HHS – Health and Human Services.

### Annual trends for PD-related mortality stratified by sex

**Figure 4.**
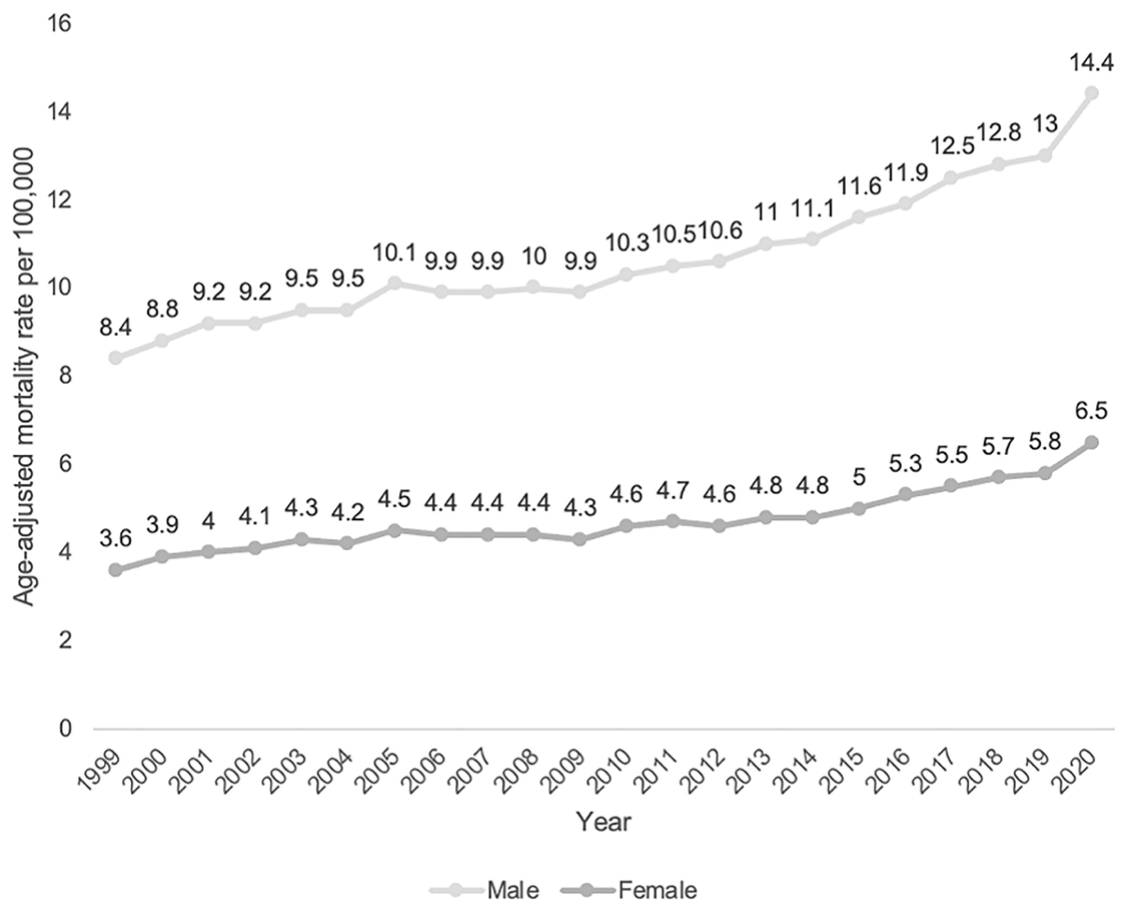
Crude mortality rate from 1999–2020 stratified by sex.

**Figure 5.**
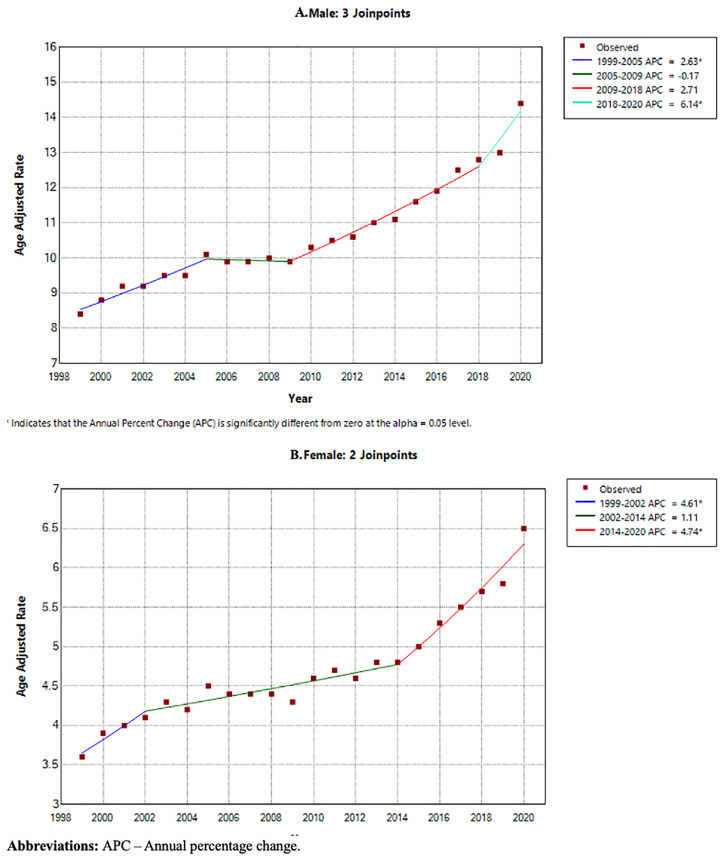
Joinpoint regression analysis by year stratified by sex: **A**. Male, **B**. Female.

Male sex had a consistently higher AAMR (10.9 per 100,000 95% CI 10.8-0.9) than female sex (4.8 per 100,000 95% CI 4.7-4.8) across the study period (shown in Table 1). Using Joinpoint regression analysis, the APC in AAMR for males was 2.63 (95% CI 0.92-4.33) from 1999–2005, which then decreased to -0.17 (95% CI -1.61-3.91) from 2005–2009, increased to 2.71 (95% CI -0.36-3.48) from 2009–2018 and increased further to 6.14 (95% CI 2.93-7.90) from 2018–2020. The APC in AAMR for females was 4.61 (95% CI 2.01-9.44) from 1999–2002, 1.11 (95% CI -1.27-1.54) from 2002–2014, then 4.74 (95% CI 3.41-7.56) from 2014–2020 (shown in Figure 4 and Figure 5).

### Annual trends for PD-related mortality stratified by race/ethnicity

**Figure 6.**
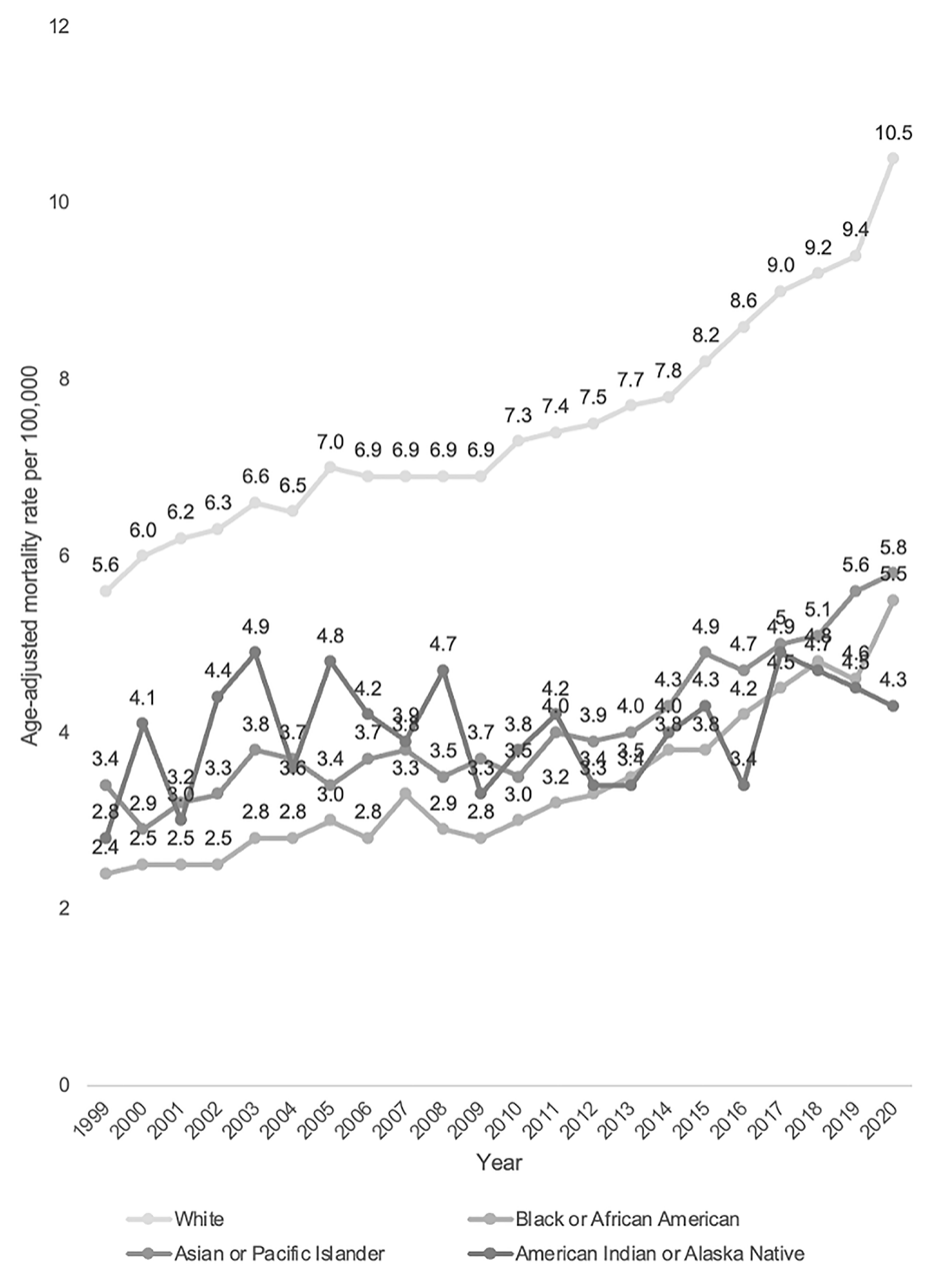
Crude mortality rate from 1999–2020 stratified by race.

**Figure 7.**
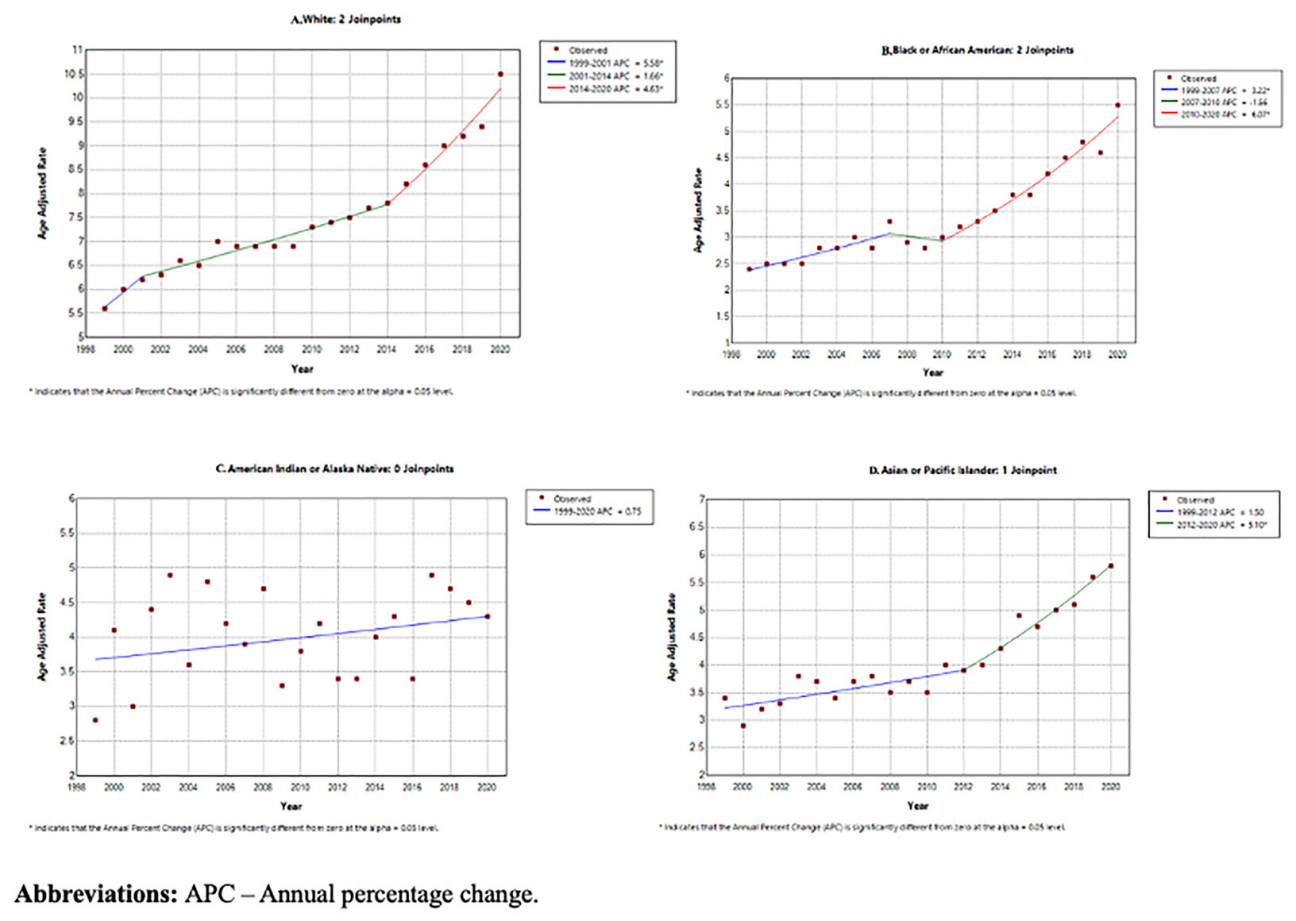
Joinpoint regression analysis by year stratified by sex: **A**. White, **B**. Black/African American, **C**. American Indians/Alaska Natives, **D**. Asians/Pacific Islanders.

White patients had consistently higher AAMR (7.6 per 100,000 95% CI 7.6-7.6) than American Indians/Alaska Natives (4.4 per 100,000 95% CI 4.3-4.4), Asians/Pacific Islanders (4.1 per 100,000 95% CI 3.9-4.3) and Black/African Americans (3.4 per 100,000 95% CI 3.5-3.6) (shown in Table 1). Using Joinpoint regression analysis, the APC in AAMR for white patients was 5.58 (95% CI 2.16-8.28) from 1999–2001, 1.66 (95% CI 0.33-1.98) from 2001–2014 and 4.63 (95% CI 3.64-6.23) from 2014 to 2020. The APC in AAMR for American Indians/Alaska Natives was 0.75 (95% CI -0.58-2.11) from 1999–2020. The APC in AAMR for Asians/Pacific Islanders was 1.50 (95% CI -1.05-2.37) from 1999–2012 and 5.10 (95% CI 3.29-11.18) from 2012–2020. The APC in AAMR for Black/African Americans was 3.22 (95% CI 1.84-7.93) from 1999–2007, reducing to -1.56 (95% CI -3.84-8.46) from 2007–2010, then increasing to 6.07 (95% CI 2.72-8.47) from 2010–2020 (shown in Figure 6 and Figure 7).

### Census region and census division level data

The Midwest census region had the highest AAMR (7.7 per 100,000 95% CI 7.6-7.7), followed by the West (7.4 per 100,000 95% CI 7.4-7.5), South (7.2 per 100,000 95% CI 7.1-7.2) and Northeast (6.2 per 100,000 95% CI 6.1-6.2). The census division with the highest AAMR was Mountain (8.1 per 100,000 95%CI 8.0-8.2), followed by West North Central (8.0 per 100,000 95% CI 7.9-8.1), East North Central (7.5 per 100,000 95% CI 7.5-7.6), West South Central (7.5 per 100,000 95% CI 7.5-7.6), East South Central (7.2 per 100,000 95% CI 7.1-7.3), Pacific (7.2 per 100,000 95% CI 7.1-7.2), South Atlantic (7.0 per 100,000 95% CI 6.9-7.0), New England (6.8 per 100,000 95% CI 6.7-6.9) and Middle Atlantic (6.0 per 100,000 95% CI 5.9-6.0) (shown in Table 2).

### Health and Human Services region level data

The top 3 health and human services (HHS) regions with the highest AAMR was region 10 (Alaska, Idaho, Oregon, Washington) (8.4 per 100,000 95% CI 8.3-8.5), followed by region 8 (Colorado, Montana, North Dakota, South Dakota, Utah and Wyoming) (9.1 per 100,000 95% CI 8.0-8.2) and region 7 (Iowa, Kansas, Missouri, Nebraska) (7.8 per 100,000 95% CI 7.7-7.9) (shown in Table 2).

### State level mortality rate

The 3 states with the highest AAMR were Utah (10.1 per 100,000 95% CI 9.7-10.4), followed by Idaho (9.0 per 100,000 95% CI 8.7-9.3) and Minnesota (8.9 per 100,000 95% CI 8.8-9.1). The 3 states with the lowest AAMR over the study period were New York (4.7 per 100,000 95% CI 4.7-4.8), followed by the District of Columbia (4.9 per 100,000 95% CI 4.5-5.3) and Connecticut (6.2 per 100,000 95% CI 6.0-6.4) (shown in Table 3 and Figure 3).

**Table 3.** Mortality rate stratified by state.

State	AAMR [95%CI]
**Alabama**	7.5 [7.3-7.6]
**Alaska**	7.1 [6.5-7.7]
**Arizona**	8.1 [7.9-8.2]
**Arkansas**	6.5 [6.3-6.7]
**California**	6.8 [6.7-6.8]
**Colorado**	8.2 [8.0-8.4]
**Connecticut**	6.2 [6.0-6.4]
**Delaware**	6.7 [6.4-7.1]
**District of Columbia**	4.9 [4.5-5.3]
**Florida**	6.7 [6.6-6.7]
**Georgia**	7.2 [7.1-7.3]
**Hawaii**	6.4 [6.1-6.6]
**Idaho**	9.0 [8.7-9.3]
**Illinois**	7.4 [7.3-7.5]
**Indiana**	7.8 [7.7-8.0]
**Iowa**	7.7 [7.5-7.8]
**Kansas**	8.5 [8.3-8.7]
**Kentucky**	7.3 [7.1-7.4]
**Louisiana**	7.3 [7.1-7.5]
**Maine**	8.1 [7.8-8.4]
**Maryland**	7.1 [6.9-7.2]
**Massachusetts**	6.6 [6.5-6.7]
**Michigan**	7.5 [7.3-7.6]
**Minnesota**	8.9 [8.8-9.1]
**Mississippi**	6.4 [6.2-6.6]
**Missouri**	7.3 [7.2-7.5]
**Montana**	7.3 [6.9-7.6]
**Nebraska**	8.5 [8.2-8.7]
**Nevada**	6.3 [6.1-6.6]
**New Hampshire**	7.7 [7.3-8.0]
**New Jersey**	6.5 [6.4-6.6]
**New Mexico**	8.1 [7.9-8.4]
**New York**	4.7 [4.7-4.8]
**North Carolina**	7.1 [7.0-7.3]
**North Dakota**	7.1 [6.7-7.5]
**Ohio**	7.5 [7.4-7.6]
**Oklahoma**	7.1 [7.0-7.3]
**Oregon**	8.4 [8.2-8.6]
**Pennsylvania**	7.3 [7.2-7.4]
**Rhode Island**	6.6 [6.3-6.9]
**South Carolina**	7.3 [7.1-7.4]
**South Dakota**	6.9 [6.5-7.2]
**Tennessee**	7.4 [7.2-7.5]
**Texas**	7.8 [7.8-7.9]
**Utah**	10.1 [9.7-10.4]
**Vermont**	8.3 [7.9-8.8]
**Virginia**	7.4 [7.2-7.5]
**Washington**	8.4 [8.2-8.5]
**West Virginia**	6.7 [6.5-6.9]
**Wisconsin**	7.8 [7.7-7.9]
**Wyoming**	6.5 [6.0-6.9]

**Abbreviations:** AAMR – Age adjusted mortality rate; CI – Confidence interval.

### Place of death

Of all patient deaths (515,884), the most common place of death was a nursing home/long term care (45.7%), followed by the decedent’s home (28.3%), medical facility – inpatient (12.7%), other place of death (6.0%), hospice facility (5.4%), medical facility – outpatient/emergency room (1.5%), unknown place (0.2%) and medical facility – death on arrival (0.2%) (shown in Table 4).

**Table 4.** Location of death.

Location	Deaths	Percentage of Total Deaths, %
Medical Facility - Inpatient	65513	12.7
Medical Facility - Outpatient or ER	7659	1.5
Medical Facility - Dead on Arrival	844	0.2
Medical Facility - Status unknown	169	0.0
Decedent's home	145882	28.3
Hospice facility	28031	5.4
Nursing home/long term care	235672	45.7
Other	31047	6.0
Place of death unknown	1067	0.2

**Abbreviations:** ER – Emergency room.

## Discussion

To the best of our knowledge, this is the first study to investigate the trends in PD-related mortality in the US. There are several important findings. Firstly, PD-related mortality in the US has steadily increased over a 20 year period, with an AAMR of 5.3 per 100,000 in 1999 compared to 9.8 per 100,000 in 2020. Secondly, there are significant geographical differences in PD-related mortality, with Midwest regions associated with the highest mortality rate and top 3 states with highest AAMR being Utah, Idaho and Minnesota. Finally, there are significant differences in demographic trends in mortality, where older age, male sex and white race associated with higher AAMRs over the 20 year study period.

The average age of the population is increasing, and along with improved management of acute and chronic conditions, patients are living longer placing them at risk of chronic diseases ^19^. Age is strongly associated with the development of PD, with a later age of diagnosis of PD linked to more severe motor deficit, postural instability and rapid disease progression ^20^. Ageing alters body homeostasis through its effect on cellular processes causing mitochondrial dysfunction, free radical production and oxidative stress, predisposing to neurodegeneration of the substantia nigra ^21^. Furthermore, the iron content of the substantia nigra increases with age and is linked to their decrease in function ^22^. The present analysis demonstrated that increasing age was associated with an increased AAMR. Previous studies have elicited this finding, with some displaying better overall prognosis and response to pharmacological treatment such as Levodopa therapy in younger patients ^23, 24^.

This study reports sex differences in PD-related mortality. There are no studies that have analysed temporal trends in sex-related disparities in PD patients. Previous studies have reported an increased prevalence of PD in males, and increased likelihood of PD mortality in females ^6, 7^. Increased prevalence in males may be due to findings of lack of protective effects of male gonadal factors, in contrast to female gonadal factors which are suspected to be protective for PD ^6, 7^. Females have a higher likelihood of overall and earlier disease course-related mortality from PD compared to males ^25^. This could be due to a later age of diagnosis given the aforementioned protective factors, which is associated with more severe symptoms, lower responsiveness to guideline indicated treatment.

The present study reports important racial differences in PD-related mortality, with white race associated with the highest AAMR. This could be because PD is most prevalent in white populations, possibly due to genetic susceptibility ^5^. Several studies state black patients have a higher risk of mortality than white patients ^26– 28^. Common reasons for poor outcomes in black patients could be multifactorial. There are several social determinants of health that could mediate outcomes in black populations which have been demonstrated across many different studies involving the US, including geographic location, socioeconomic status and access to healthcare ^29– 31^. State-level disparities in the context of US healthcare in particular could be due to varying levels of accessibility to services and affordability ^32^. Furthermore, certain regions of the US such as the Midwest have a higher proportion of individuals who are holder and white race ^33^. This could explain increased AAMR in the Midwest region and associated states.

Admissions to hospital in patients with PD in the US have been steadily increasing. An analysis using the National Inpatient Sample demonstrated that from 2002–2016, admissions in patients with PD rose from 212,070 to 312,980 ^34^. Mortality as an inpatient during this time decreased from 4.8% in 2002–2004 to 3.6% in 2014–2016 ^34^. In the present study, PD-related AAMR increased over the study period, suggesting patients with PD are likely die outside of hospital, as this analysis also demonstrates. Previous studies have demonstrated a similar trend. An analysis of the World Health Organisation Mortality Database from 1994–2019 showed that mortality rate in 1994 was 1.76 per 100,000, rising to 5.67 per 100,000 in 2019. Mortality rate was higher in males and in European countries and the United States ^3^. Another study similar to the present study where AAMR from PD increased from 5.4 per 100,000 to 8.8 per 100,000 in a 20 year period ^35^. The present study observed a similar trend, with AAMR 5.3 per 100,000 in 1999 and 9.8 per 100,000 in 2020.

There are several important clinical implications of this study. This study demonstrates that PD is present as an underlying cause in a significant number of deaths in the US. Knowledge of trends in PD-related mortality is fundamental to building healthcare infrastructure around the growing needs of the increasing population living with PD. This study reports significant differences in demographic trends in PD-related mortality. Increasing age, male sex and white race were associated with the highest AAMR. This supports increased focus into the underlying reasons for these disparities and research into how these disparities can be addressed and managed.

There are several limitations to this study inherent to the dataset used. Firstly, routinely collected data from death certificates may be subject to coding inaccuracies, although there is no reason to believe this would bias findings in one direction or another, it may decrease precision. Secondly, data on overall prevalence, pharmacotherapy, co-morbidities and procedures that could have mediated outcomes are not available on the dataset, and therefore, we were not able to consider the rate of deaths allowing for the underlying rate of disease or the pathway from diagnosis to death. Finally, with this dataset we cannot ascertain the exact primary cause of death (e.g. cardiovascular disease, respiratory disorders, trauma), although arguably this is not the question at hand if PD is underlying this primary cause of death. This study records PD as an underlying cause of death where it is recorded on the death certificate of the deceased patient.

Future research could investigate underlying reasons behind disparities in mortality. Given the present study confirms populations with the highest prevalence of PD (older, male and white), also have the highest AAMR, further work must be completed to determine underlying reasons behind poor outcomes in female sex and non-white groups discovered in other studies. Knowledge of the factors contributing to poor outcomes could be fundamental to identify at-risk individuals. These individuals can be targeted for risk factor optimisation with the aim to reduce downstream adverse outcomes. Further research could also investigate the interplay between the aforementioned social determinants, age of diagnosis, onset of symptoms, comorbidities and how these impact demographic differences in PD-related mortality.

## Conclusions

In conclusion, there are significant differences in demographic trends in PD-related mortality in the US. PD accounts for a greater number of deaths per head of population at older ages, in males, people of white race and in the Midwest. Knowledge of these trends is important to build healthcare services around this growing population. Further research must focus on addressing these differences to optimise management and development of appropriate healthcare infrastructure around this growing, at-risk population.

## Statements and declarations

### Ethics and consent

This study did not require ethical approval from an institutional review board. CDC-WONDER is an publicly available, anonymised dataset. made from routinely collected death certificate data. All data are covered by the provisions of the Public Health Service Act (42 U.S.C. 242m(d)).

## Data Availability

All data available in this article is publicly accessible from the database website https://wonder.cdc.gov.
