# Supplementary material for: Temporal Trends in Parkinson’s Disease Related Mortality from 1999-2020: A National Analysis
Source: NIHR Open Res. 2024 Sep 13;4:50. [Version 1] doi: 10.3310/nihropenres.13623.1 (PMC11739699; doi:10.3310/nihropenres.13623.1)
Supplement: Peer review history

# Reviewer report: Reviewer response for version 1

**DOI:** 10.3310/nihropenres.14792.r33590

**Published:** 2025-01-16

**Authors:** Jooyoung Lee

**Affiliations:** 1 Chung-Ang University, Seoul, South Korea

1. Rong et al. (2021) conducted similar analyses to examine temporal trends in Parkinson’s disease mortality in the USA from 1999 to 2019 using data from the National Vital Statistics System (NVSS) of the National Center for Health Statistics (NCHS) and the Centers for Disease Control and Prevention (CDC). They also calculated the age-adjusted mortality rate each year from 1999 to 2019, stratified by age, sex, ethnicity, and region. I wonder what new insights this manuscript provides.

Rong, S., Xu, G., Liu, B., Sun, Y., Snetselaar, L. G., Wallace, R. B., ... & Bao, W. (2021). Trends in mortality from Parkinson disease in the United States, 1999–2019. *Neurology*, *97*(20), e1986-e1993.

 2. Can you provide more details about the joinpoint regression models? How was the statistical significance of a change in trend assessed? Have you specified the minimum and maximum number of joinpoints?

 3. In the Discussion section, the authors noted that the higher mortality rate among White patients compared to Black patients was due to the higher prevalence of Parkinson’s disease (PD) in White individuals. You may consider presenting prevalence values of PD by race/ethnicity from other literature to support this point.

Is the work clearly and accurately presented and does it cite the current literature?

Yes

If applicable, is the statistical analysis and its interpretation appropriate?

Yes

Are all the source data underlying the results available to ensure full reproducibility?

Yes

Is the study design appropriate and is the work technically sound?

Yes

Are the conclusions drawn adequately supported by the results?

Yes

Are sufficient details of methods and analysis provided to allow replication by others?

Partly

Reviewer Expertise:

Biostatistics, Epidemiology

I confirm that I have read this submission and believe that I have an appropriate level of expertise to confirm that it is of an acceptable scientific standard, however I have significant reservations, as outlined above.

## References

[1] : Trends in Mortality From Parkinson Disease in the United States, 1999-2019. *Neurology* .2021;97(20) : 10.1212/WNL.0000000000012826 e1986-e1993 10.1212/WNL.0000000000012826 34706971

# Reviewer report: Reviewer response for version 1

**DOI:** 10.3310/nihropenres.14792.r34189

**Published:** 2025-01-06

**Authors:** Lawrence T Dauer

**Affiliations:** 1 Memorial Sloan Kettering Cancer Center, New York, USA

Article reports on age-adjusted mortality rates of Parkinson's Disease using the CDC-WONDER underlying COD database from 1999-2020 and reports a near doubling of the AAMR over that period and that older age, males, white race, and Midwest regions had highest AAMRs. Note that this is not the first study over this time period that has utilized the same database. In the present form, it is not clear what this evaluation provides that is new or original. 

 Several comments/suggestions for Improvement:

 1. Census region, HHS region, and State level mortality results could be improved if there were adjustments based on race and age demographics for those regions (as pointed out by the authors Midwest has older and more generally white). Census data over time could be utilized for those adjustments. An adjusted evaluation would be more informative when evaluating regional differences. If such an evaluation were performed, it would be a unique addition to the literature that already exists over this time period. In addition a rural vs urban breakdown would be helpful to compare with other previously reported results.

 2. Page 7 of 15 - as this is not the first study to investigate trends in PD-related mortality in the US (example Ref 35 - and others noted below), should remove this sentence.

 3. Page 7 of 15 - other studies have evaluated sex-related differences ( example Ref 35 and others) and they should be compared with.

 4. Page 7 of 15 - spelling error for "holder" meaning "older".

 5. Figures 2 and 6 should be enlarged, difficult to read. 

 6. Several other studies should be referenced and discussed and a clear distinction made between this study and those earlier reported results. In fact, Ref 35, Rong et al should be compared with as it is not clear what is different in this study vs that study. In addition, see other examples:

 Tharwani et al 2024 - https://www.prd-journal.com/article/S1353-8020(24)01122-2/abstract

 and a systematic review of global trends from Lancet

 Zhu et al 2024 (Ref-1) - https://www.thelancet.com/journals/lanhl/article/PIIS2666-7568(24)00094-1/fulltext

Is the work clearly and accurately presented and does it cite the current literature?

No

If applicable, is the statistical analysis and its interpretation appropriate?

Yes

Are all the source data underlying the results available to ensure full reproducibility?

Yes

Is the study design appropriate and is the work technically sound?

Yes

Are the conclusions drawn adequately supported by the results?

Partly

Are sufficient details of methods and analysis provided to allow replication by others?

Yes

Reviewer Expertise:

Cognitive related outcome epidemiology.

I confirm that I have read this submission and believe that I have an appropriate level of expertise to confirm that it is of an acceptable scientific standard, however I have significant reservations, as outlined above.

## References

[1] : Temporal trends in the prevalence of Parkinson's disease from 1980 to 2023: a systematic review and meta-analysis. *Lancet Healthy Longev* .2024;5(7) : 10.1016/S2666-7568(24)00094-1 e464-e479 10.1016/S2666-7568(24)00094-1 38945129

# Reviewer report: Reviewer response for version 1

**DOI:** 10.3310/nihropenres.14792.r34191

**Published:** 2024-12-31

**Authors:** Jenny Sassone

**Affiliations:** 1 IRCCS San Raffaele Scientific Institute, Vita-Salute San Raffaele University,, Milan, Italy

This study analyzes the demographic trends in

 PD-related mortality in the United States. The data are from CDC-WONDER and refer to the time interval January 1999 - December 2020.

 The work clearly and accurately presents and cites the literature.

 The statistical analysis and its interpretation are appropriate

 If possible, it would be useful and interesting to add a section about the cause of death (cancer, depression, ischemic heart disease, stroke…….).

 This section would give a more complete view of the results.

Is the work clearly and accurately presented and does it cite the current literature?

Yes

If applicable, is the statistical analysis and its interpretation appropriate?

Yes

Are all the source data underlying the results available to ensure full reproducibility?

Yes

Is the study design appropriate and is the work technically sound?

Yes

Are the conclusions drawn adequately supported by the results?

Yes

Are sufficient details of methods and analysis provided to allow replication by others?

Yes

Reviewer Expertise:

parkinson's disease

I confirm that I have read this submission and believe that I have an appropriate level of expertise to confirm that it is of an acceptable scientific standard.
